# Assessment of Choroidal Thickness Inside and Outside of Vascular Arcade in Diabetic Retinopathy Eyes Using Spectral-Domain Optical Coherence Tomography

**DOI:** 10.1038/s41598-019-47351-w

**Published:** 2019-07-25

**Authors:** Kyeong Do Jeong, Jae Yong Park, Bo Na Kim, Jae Suk Kim, Min Ji Kang, Je Hyung Hwang

**Affiliations:** 1 Asan-si Health Care Center, Chungcheongnam-do, Seoul, South Korea; 2 Department of Ophthalmology, Sanggye Paik Hospital, Inje University, Seoul, South Korea; 3 Mirae Eye Clinic, Gwangmyeong-si, Seoul, South Korea

**Keywords:** Uveal diseases, Retinal diseases

## Abstract

This study aimed to characterise the distribution of choroidal thickness (CT) in diabetic retinopathy eyes, inside and outside of the vascular arcade, as well as at the fovea, using spectral-domain optical coherence tomography (OCT). Forty-nine healthy eyes, 80 diabetic retinopathy (DR) eyes (59 non-proliferative diabetic retinopathy (NPDR) eyes and 21 proliferative diabetic retinopathy (PDR) eyes) were examined with OCT to obtain nine horizontal lines (far superotemporal, near superotemporal, central, near inferotemporal, far inferotemporal, far superonasal, near superonasal, near inferonasal, far inferonasal) inside and outside of the vascular arcade. Nine points were chosen in 0.5-mm intervals to calculate CT, which was measured at 81 points in each patient. In the DR group, CT decreased significantly, compared with the control group, in all nine horizontal lines except central and near inferotemporal (−29.74 to −36.97 μm, p < 0.05 for all). In the PDR group, CT decreased compared with the NPDR group, in all nine horizontal lines (−6.18 μm to −34.58 μm), but this difference was not significant. In DR eyes, an overall significant reduction of CT was observed inside and outside of the vascular arcade; CT showed a non-significant decrease in PDR eyes, compared with NPDR eyes.

## Introduction

Diabetic retinopathy (DR) is a complication of diabetes mellitus (DM) and a leading cause of blindness worldwide^1^. DR is generally thought to be caused by abnormalities of retinal microvasculature, but recent studies have shown that the choroid plays an important role in the progress of DR^2,3^. The choroid is a layer of the eye that provides 95% of the ocular blood flow; it supplies oxygen and nutrients to the outer retina, including photoreceptors and retinal pigment epithelium, and is the sole provider of blood flow to the avascular fovea^4^. This function of the choroid is affected in DR, in association with choroidal vasculopathy^3,5,6^.

Many studies have been conducted to investigate the relationship between DR and choroidal thickness (CT). In the past, CT was measured using ultrasonography, but this method was characterised by an inability to measure the exact thickness or detect small changes^7^. In recent years, optical coherence tomography (OCT) has become the gold standard for retinal imaging, and the enhanced depth imaging (EDI) mode has enabled acquisition of images of the choroid below the retina^8^. OCT has thus been used to investigate the relationship between DR and CT. Thus far, changes have been assessed in subfoveal, parafoveal, and parapapillary CT, among DR patients, regardless of severity. In the present study, we used the EDI mode of spectral domain-OCT to characterise changes in CT in the periphery of the macula, as well as outside of the vascular arcade, in DR patients. Moreover, we assessed variations in peripheral CT according to the degree of DR severity.

## Results

This study included 49 subjects in the control group (26 men, 23 women), 59 in the non-proliferative DR (NPDR) group (30 men and 29 women), and 21 in the proliferative DR (PDR) group (10 men and 11 women). The mean ages of subjects in the three groups were 59.67 ± 10.01 years, 61.20 ± 11.11 years, and 63.29 ± 9.40 years, respectively; age did not significantly differ among the groups (p = 0.405) (Table 1). Intraclass correlation coefficient between the two independent observers was 0.990 (P < 0.001).

**Table 1:** Baseline characteristics.

	Control group	NPDR group	PDR group
Number of subjects	49	59	21
Sex (M/F)	26/23	30/29	10/11
Age (years, mean ± SD)	59.67 ± 10.01	61.20 ± 11.11	63.29 ± 9.40

M, male; F, female; SD, standard deviation; NPDR, non-proliferative diabetic retinopathy; PDR, proliferative diabetic retinopathy.

**Figure 1:**
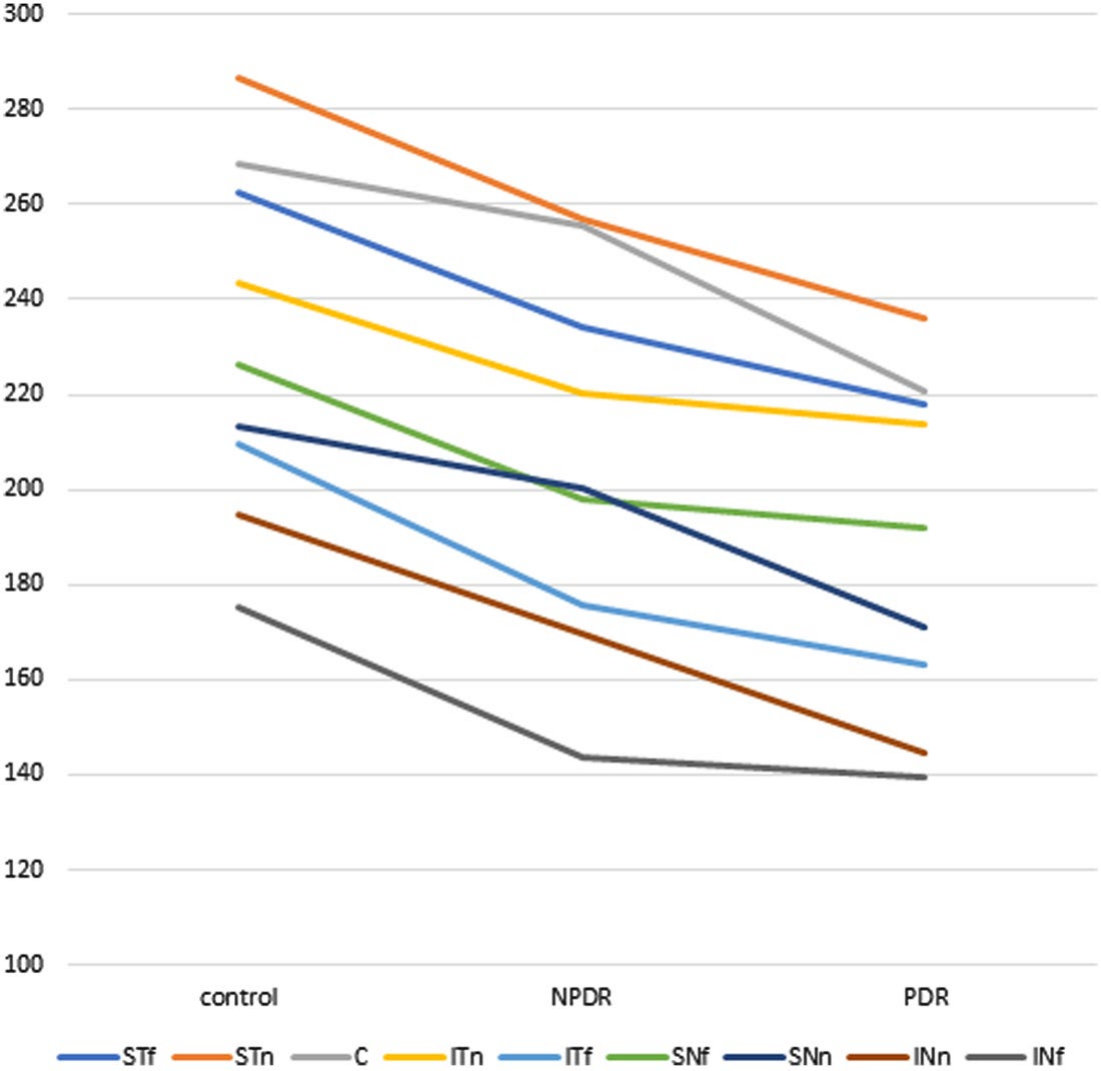
Changes in choroidal thickness with the progression of diabetic retinopathy in nine horizontal lines. NPDR, non-proliferative diabetic retinopathy; PDR, proliferative diabetic retinopathy; STf, far superotemporal; STn, near superotemporal; Cf, central; ITn, near inferotemporal; ITf, far inferotemporal; SNf, far superonasal; SNn, near superonasal; INn, near inferonasal; INf, far inferonasal.

Comparison of CT between the control and DR groups revealed that CT significantly decreased in all nine horizontal lines, except centre (Cf) and near inferotemporal (ITn). Notably, the CT also decreased in Cf and ITn, but these differences were not statistically significant (p = 0.092, p = 0.051) (Table 2). Comparison of NPDR and PDR groups (i.e., subgroups within the DR group) revealed that the CT of the PDR group decreased in all nine horizontal lines, but these differences were not statistically significant (Table 3). Comparison of the control, NPDR, and PDR groups showed that CT continuously decreased in all nine horizontal lines, in accordance with the progression of DR (Fig. 1).

**Table 2:** Comparison of choroidal thickness between control and DR groups in nine horizontal lines.

Horizontal lines	Control	DR	p-value
STf	262.40 ± 45.98	229.93 ± 65.18	0.001
STn	286.60 ± 50.71	251.37 ± 83.23	0.003
Cf	268.73 ± 49.52	246.26 ± 99.78	0.092
ITn	243.56 ± 55.91	218.45 ± 89.04	0.051
ITf	209.40 ± 55.76	172.43 ± 64.42	0.001
SNf	226.22 ± 50.44	196.48 ± 62.12	0.005
SNn	213.21 ± 50.06	192.46 ± 67.18	0.048
INn	194.78 ± 51.50	163.10 ± 66.90	0.003
INf	175.40 ± 48.10	142.49 ± 47.03	<0.001

In control and DR columns, values are mean ± standard deviation (in μm).

DR, diabetic retinopathy; STf, far superotemporal; STn, near superotemporal; Cf, central; ITn, near inferotemporal; ITf, far inferotemporal; SNf, far superonasal; SNn, near superonasal; INn, near inferonasal; INf, far inferonasal.

**Table 3:** Comparison of choroidal thickness between NPDR and PDR groups in nine horizontal lines.

Horizontal lines	NPDR	PDR	p-value
STf	234.24 ± 62.10	217.83 ± 73.43	0.325
STn	256.75 ± 85.79	236.24 ± 75.45	0.335
Cf	255.34 ± 105.94	220.76 ± 76.50	0.174
ITn	220.08 ± 88.09	213.88 ± 93.69	0.786
ITf	175.79 ± 56.07	163.01 ± 84.54	0.525
SNf	198.11 ± 59.49	191.92 ± 70.34	0.721
SNn	200.16 ± 64.86	170.84 ± 70.43	0.086
INn	169.71 ± 65.50	144.52 ± 68.88	0.139
INf	143.63 ± 47.38	139.29 ± 47.01	0.719

In NPDR and PDR columns, values are mean ± standard deviation (in μm).

NPDR, non-proliferative diabetic retinopathy; PDR, proliferative diabetic retinopathy; STf, far superotemporal; STn, near superotemporal; Cf, central; ITn, near inferotemporal; ITf, far inferotemporal; SNf, far superonasal; SNn, near superonasal; INn, near inferonasal; INf, far inferonasal.

Comparison of the CT reduction rate based on the progress of DR for each of the nine horizontal lines showed that the CT reduction rate was greater for the far lines than for the near lines; however, there was no particular tendency in the comparison between NPDR and PDR groups. The NPDR group showed a reduction of 5–18.1% in CT, relative to that of the control group, while the PDR group showed a reduction of 3–14.8% in CT, relative to that of the NPDR group (Table 4).

**Table 4:** Comparison of reduction rate of choroidal thickness with progression of diabetic retinopathy in nine horizontal lines.

	Control/NPDR	NPDR/PDR	Control/PDR
STf	0.893	0.930	0.830
STn	0.896	0.920	0.824
Cf	0.950	0.865	0.822
ITn	0.904	0.972	0.878
ITf	0.839	0.927	0.778
SNf	0.876	0.969	0.848
SNn	0.939	0.854	0.801
INn	0.871	0.852	0.742
INf	0.819	0.970	0.794

NPDR, non-proliferative diabetic retinopathy; PDR, proliferative diabetic retinopathy; STf, far superotemporal; STn, near superotemporal; Cf, central; ITn, near inferotemporal; ITf, far inferotemporal; SNf, far superonasal; SNn, near superonasal; INn, near inferonasal; INf, far inferonasal.

## Discussion

Previous studies measured the CT around the fovea to determine its correlation with DR. Endo *et al*.^9^, Rewbury *et al*.^10^, and Eliwa *et al*.^11^ only measured subfoveal CT. In contrast, Tavares Ferreira *et al*.^12^, Unsal *et al*.^13^, Tavares Ferreira *et al*.^14^, and Kim *et al*.^15^ measured CT within 1.5 mm from the fovea. Regatieri *et al*.^16^ measured CT within 2.5 mm from the fovea, while Sheth *et al*.^17^ measured CT within 3 mm from the fovea. Lains *et al*.^18^ performed a 12-mm × 9-mm volume scan using swept-source OCT, and Esmaeelpour *et al*.^19^ observed the CT change in a 36° × 36° field around the fovea using three-dimensional 1060-nm OCT. Notably, all of these preceding studies observed CT changes inside the vascular arcade. The present study is clinically meaningful because—to the best of our knowledge—it is the first to investigate CT changes outside of the vascular arcade in DR patients.

The overall CT distribution in DR patients is similar to that in normal subjects. Park *et al*.^20^ reported that the CT in normal subjects is thickest near the superotemporal aspect of the temporal area and becomes thinner toward the periphery. In the nasal area, the CT is thickest near the superonasal aspect and becomes thinner in the caudal direction. In the present study, both NPDR and PDR groups exhibited a normal CT distribution, similar to that of previous studies. Previous studies have shown that the central CT becomes thinner in patients with DR than in normal subjects. Querques *et al*.^21^ reported that the subfoveal CT decreased more in patients who had diabetes with retinopathy than in those who had diabetes without retinopathy. However, there is controversy regarding changes in CT around the macula, depending on the progress of DR. Vujosevic *et al*.^22^ reported that macular and peripapillary CTs decreased as DR progressed. Moreover, Regataeri *et al*.^16^ reported that the PDR and diabetic macular edema (DME) groups showed a significant reduction in CT compared to that of the control group, whereas the NPDR group did not show a significant difference in CT compared to that of the control group. However, Shen *et al*.^14^ reported that the CTs measured with cirrus OCT in patients with mild to moderate NPDR significantly decreased when compared to those of the control group. Kim *et al*.^15^ and Rewbury *et al*.^10^ reported that the CT increased from moderate-severe NPDR to untreated PDR, and that the DME group had thicker CT than did the non-DME group. In this study, when the DR group was divided into two groups (NPDR and PDR), the CT decreased as DR progressed to PDR; however, there was no significant difference in CT between NPDR and PDR groups.

The results of the present study confirm that CT decreases in the central region of the choroid, and reveal that CT decreases outside of the vascular arcade; this latter finding has not been described in previous reports. In the PDR group, the CT decreased by approximately 12.2–25.8%, compared to the CT in the control group. This suggests that changes in CT due to DR are reduced throughout the choroid, as well as in the subfoveal and parafoveal portions of the choroid. It is reasonable to hypothesise that the reduction of choroidal circulation due to DR is the underlying cause of a reduction in peripheral macular CT in patients with DR. Previous histological studies have reported increased blood vessel tortuosity, local vasodilatation and stenosis, formation of nonperfusion area, and loss of choriocapillaris in DM patients^3,23–25^. In the prior studies, the authors also reported the use of laser doppler flowmetry to observe that choroidal changes in choriocapillaris caused choroidal changes in early choroidal vessels, rather than in large choroidal vessels. Indocyanine green angiography showed that choroidal vascular filling delay increases with progression of DR; in patients with DME, choroidal circulation showed further reduction^26–28^. These changes occurred in the vascular arcade and throughout the choroid (i.e., outside of the vascular arcade). Therefore, as shown in the present study, the peripheral CT will be reduced with the progression of DR. In the future, further studies are needed regarding peripheral choroidal blood flow.

There were some limitations in this study. First, a group of patients with DME was not clearly defined. Previous studies have shown that subfoveal CT depends on the presence of DME. Kim *et al*.^15^ reported that the DME group had increased subfoveal CT, compared to the non-DME group; furthermore, Sheth *et al*.^17^ reported that subfoveal CT increased in DM patients without macular ischemia. However, Unsal *et al*.^13^ and Nagaoka *et al*.^26^ reported that the DME group had lower CT than the control group. Understanding of DR will be enhanced by more thorough characterisation of the correlation between peripheral CT and the presence of DME. Furthermore, a group of patients who had DM without DR was not considered separately, in contrast to the approach of other studies. Shen *et al*.^14^ reported that the DM without DR group exhibited reduced subfoveal CT, compared to that in the control group. Because DM can affect the choroid regardless of the presence of DR, further studies regarding peripheral choroid may facilitate understanding of the effect of DM on the choroid. The duration of diabetes and diabetic retinopathy were not confirmed by retrospective review of medical records. The choroidal thickness may vary according to the duration of the disease, so future studies will need to reflect this. In addition, studies are needed regarding changes in CT that occur in mild, moderate, and severe stages of NPDR.

In the present study, we did not examine the relationship between CT and blood glucose levels, including HbA1c. Unsal *et al*.^13^ argued that HbA1c and central CT have a weak-moderate negative correlation. Thus, further research is needed to clarify the relationship between blood glucose level and peripheral CT. Spectral-domain OCT can be used to measure central and peripheral choroidal thickness outside the vascular arcade. Compared to normal subjects, CT decreases in both central and peripheral areas in patients with DR; notably, CT tends to decrease in accordance with the progression of DR.

## Methods

This was a retrospective review of medical records, based on data acquired at Sanggye Paik Hospital during the period from June 2012 to December 2012. Data acquisition and analysis were approved by the institutional review board of Inje University, and the institutional review board waived the requirement for informed consent because this study constituted a reanalysis of data from previously approved studies. This study was performed in accordance with the Declaration of Helsinki.

### Subjects

All recruited patients underwent dilation and then completed the following examinations: funduscopic examination, funduscopic photography, funduscopic fluorescent photography, and OCT with a slit-lamp microscope. The degree of DR was graded in accordance with the Early Treatment of Diabetic Retinopathy Study grading system. The patient group consisted of adults with DR who were over the age of 40 years. The control group also consisted of adults over the age of 40 years, because age can affect CT. Exclusion criteria were as follows: (1) a history of laser photocoagulation or intravitreal injection (anti-vascular endothelial growth factor and/or steroid treatment) or retinal surgery; (2) the presence of more than 3 dioptres of myopia; (3) the presence of macular oedema; (4) a history of age-related macular degeneration, choroidal neovascular membranes, retinal vein occlusion, or other retinal disorders; (5) a history of optic neuropathy, including glaucoma; (6) a history of any neurodegenerative disease known to affect retinal nerve fibre layer thickness; (7) the presence of systemic hypertension (blood pressure >140/90 mmHg); and (8) difficulty in observing a subject’s retina due to opaque media, or difficulty in conducting OCT for a given subject. We studied the right eyes of all subjects. The DR group was compared with the control group that was not diagnosed with DR; in addition, the DR group was divided into the NPDR and PDR groups, based on the progress of DR.

### OCT protocols

Spectral-domain OCT (Heidelberg Engineering, Heidelberg, Germany) with EDI mode and eye-tracking system was used to capture OCT images; one skilled examiner captured images of all subjects. Images of the subjects’ eyes were obtained while subjects were gazing at superior, middle, inferior, superonasal, and inferonasal spots using a built-in fixation point system in the OCT.

A 25-horizontal line posterior pole scan (30° × 20°, 240-μm intervals) was performed while subjects were gazing at the superior, middle, and inferior spots. In the superior gazing spots, the first and thirteenth lines were selected for analysis; in the middle gazing spots, the 13th line was selected; and in the inferior gazing spots, the 25th line was selected. From the top, the lines were designated as the far superotemporal line (STf), near superotemporal line (STn), central (Cf), near inferotemporal (ITn) and far inferotemporal line (ITf).

**Figure 2:**
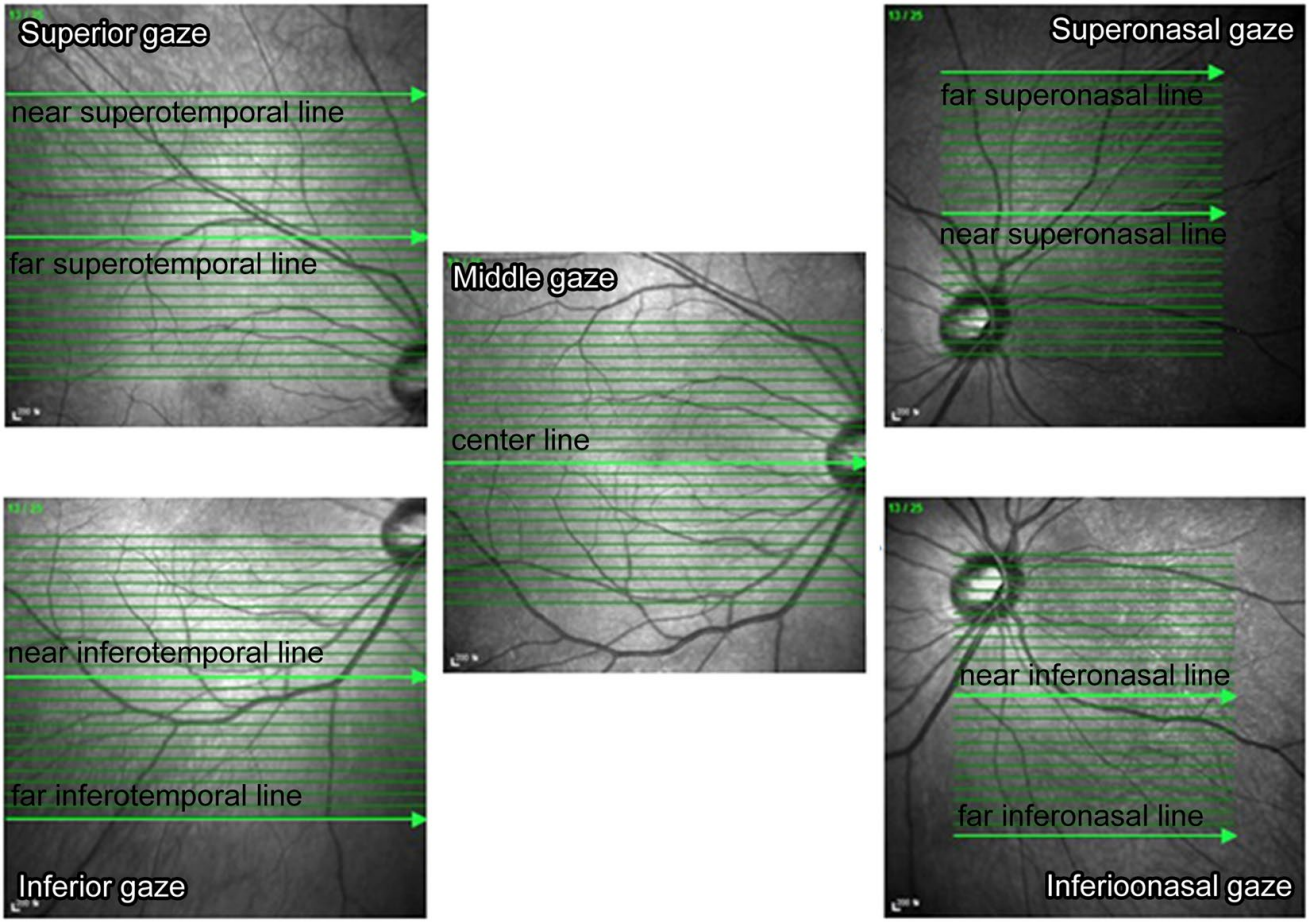
Representative model of the study on choroidal thickness in and outside of the vascular arcade using spectral-domain optical coherence tomography. Choroidal thickness was measured along far superotemporal, near superotemporal, centre, near inferotemporal, far inferotemporal, far superonasal, near superonasal, near inferonasal, and far inferonasal lines. From each of the nine lines, nine points were chosen at 0.5-mm intervals to calculate choroidal thickness.

A 25-horizontal line posterior pole scan (20° × 20°, 240-μm intervals) was performed while subjects were gazing at the superonasal and inferonasal spots. While gazing at the superonasal spot, the temporal end of the line of the scan area was manually aligned with the temporal margin of the optic disc; the 25th line was manually aligned with the inferior margin of the optic disc. Similarly, while gazing at the inferonasal spot, the temporal end of line of the scan area was manually aligned with the temporal margin of the optic disc; the first line was manually aligned with the superior margin of the optic disc. From the top, the first and 13th lines of the superonasal gazing spots, and the 13th and 15th lines of the inferonasal gazing spots, were designated as the superonasal line (SNf), near superonasal line (SNn), near inferonasal line (INn), and far inferonasal line (INf) (Fig. 2).

**Figure 3:**
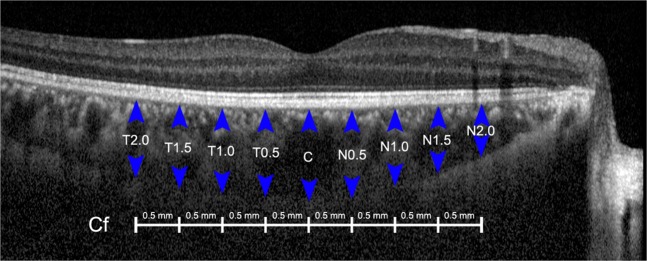
An example of choroidal thickness measurement along the centre line (Cf). Choroidal thickness was measured from the retinal pigment epithelium to the choroidoscleral junction at 0.5-mm intervals.

CT was measured manually by two skilled examiners(KDJ and JYP), using a built-in calliper within the OCT software. The average of the two measured values was used for analysis. CT was defined as the distance from the outer portion of the hyperreflective line corresponding to the retinal pigment epithelium, to the hyperreflective line between the large vessel layer of the choroid and the sclera. CTs were measured at the central point of each line, as well as at 0.5 mm, 1.0 mm, 1.5 mm, and 2.0 mm from the centre. The average CT of the nine choroidal lines was determined as the CT of each line (Fig. 3).

### Statistical analysis

For statistical analysis, Student’s t-test was performed to compare CT between groups. All analyses were performed on all nine lines mentioned above, using IBM SPSS Statistics version 25 (SPSS Inc., Chicago, IL, USA); differences were considered to be statistically significant when p < 0.05. Reliability analysis was done to calculate the intraclass correlation coefficient in order to evaluate the credibility of the CT measurement.

## Data Availability

The data are not available for public access because of patient privacy concerns, but are available from the corresponding author on reasonable request.

## References

[1] Moss SE, Klein R, Klein BE (1998). The 14-year incidence of visual loss in a diabetic population. Ophthalmology..

[2] Lee SH, Kim J, Chung H, Kim HC (2014). Changes of choroidal thickness after treatment for diabetic retinopathy. Curr. Eye. Res..

[3] Hidayat AA, Fine BS (1985). Diabetic choroidopathy. Light and electron microscopic observations of seven cases. Ophthalmology..

[4] Nickla DL, Wallman J (2010). The multifunctional choroid. Prog. Retin. Eye Res..

[5] Weinberger D (1998). Indocyanine green angiographic findings in nonproliferative diabetic retinopathy. Am. J. Ophthalmol..

[6] Shiragami C, Shiraga F, Matsuo T, Tsuchida Y, Ohtsuki H (2002). Risk factors for diabetic choroidopathy in patients with diabetic retinopathy. Graefes Arch. Clin. Exp. Ophthalmol..

[7] Coleman DJ, Lizzi FL (1979). *In vivo* choroidal thickness measurement. Am. J. Ophthalmol..

[8] Spaide RF, Koizumi H, Pozzoni MC (2008). Enhanced depth imaging spectral-domain optical coherence tomography. Am. J. Ophthalmol..

[9] Endo H (2018). Alteration of layer thickness in the choroid of diabetic patients. Clin. Exp. Ophthalmol..

[10] Rewbury R, Want A, Varughese R, Chong V (2016). Subfoveal choroidal thickness in patients with diabetic retinopathy and diabetic macular oedema. Eye (Lond.)..

[11] Eliwa TF, Hegazy OS, Mahmoud SS, Almaamon T (2017). Choroidal thickness change in patients with diabetic macular edema. Ophthalmic Surg. Lasers Imaging Retina..

[12] Tavares Ferreira J (2018). Choroidal thickness in diabetic patients without diabetic retinopathy. Retina..

[13] Unsal E (2014). Choroidal thickness in patients with diabetic retinopathy. Clin. Ophthalmol..

[14] Shen ZJ (2017). Association of choroidal thickness with early stages of diabetic retinopathy in type 2. diabetes. Int. J. Ophthalmol..

[15] Kim JT, Lee DH, Joe SG, Kim JG, Yoon YH (2013). Changes in choroidal thickness in relation to the severity of retinopathy and macular edema in type 2 diabetic patients. Invest. Ophthalmol. Vis. Sci..

[16] Regatieri CV, Branchini L, Carmody J, Fujimoto JG, Duker JS (2012). Choroidal thickness in patients with diabetic retinopathy analyzed by spectral-domain optical coherence tomography. Retina..

[17] Sheth JU, Giridhar A, Rajesh B, Gopalakrishnan M (2017). Characterization of macular choroidal thickness in ischemic and nonischemic diabetic maculopathy. Retina..

[18] Lains I (2018). Choroidal thickness in diabetic retinopathy assessed with swept-source optical coherence tomography. Retina..

[19] Esmaeelpour M (2011). Mapping choroidal and retinal thickness variation in type 2 diabetes using three-dimensional 1060-nm optical coherence tomography. Invest. Ophthalmol. Vis. Sci..

[20] Park JY, Kim BG, Hwang JH, Kim JS (2017). Choroidal thickness in and outside of vascular arcade in healthy eyes using spectral-domain optical coherence tomography. Invest. Ophthalmol. Vis. Sci..

[21] Querques G (2012). Enhanced depth imaging optical coherence tomography in type 2 diabetes. Invest. Ophthalmol. Vis. Sci..

[22] Vujosevic S, Martini F, Cavarzeran F, Pilotto E, Midena E (2012). Macular and peripapillary choroidal thickness in diabetic patients. Retina..

[23] Cao J, McLeod S, Merges CA, Lutty GA (1998). Choriocapillaris degeneration and related pathologic changes in human diabetic eyes. Arch. Ophthalmol..

[24] McLeod DS, Lutty GA (1994). High-resolution histologic analysis of the human choroidal vasculature. Invest. Ophthalmol. Vis. Sci..

[25] Fryczkowski AW, Sato SE, Hodes BL (1988). Changes in the diabetic choroidal vasculature: scanning electron microscopy findings. Ann. Ophthalmol..

[26] Nagaoka T (2004). Alteration of choroidal circulation in the foveal region in patients with type 2 diabetes. Br. J. Ophthalmol..

[27] Melancia D, Vicente A, Cunha JP, Abegao Pinto L, Ferreira J (2016). Diabetic choroidopathy: a review of the current literature. Graefes Arch. Clin. Exp. Ophthalmol..

[28] Bartsch DU, Weinreb RN, Zinser G, Freeman WR (1995). Confocal scanning infrared laser ophthalmoscopy for indocyanine green angiography. Am. J. Ophthalmol..

